# Effect of intracellular lipid accumulation in a new model of non-alcoholic fatty liver disease

**DOI:** 10.1186/1471-230X-12-20

**Published:** 2012-03-01

**Authors:** Norberto C Chavez-Tapia, Natalia Rosso, Claudio Tiribelli

**Affiliations:** 1 Fondazione Italiana Fegato- Centro Studi Fegato, AREA SCIENCE Park Basovizza, Bldg Q, Trieste, Italy; 2 Centro Studi Fegato, AREA Science Park, Bldg.Q. SS 14 km 163.5. 34012, Trieste, Italy; 3 Medica Sur Clinic and Foundation, Mexico City, Mexico

## Abstract

**Background:**

*In vitro *exposure of liver cells to high concentrations of free fatty acids (FFA) results in fat overload which promotes inflammatory and fibrogenic response similar to those observed in patients with Non-Alcoholic Fatty Liver Disease (NAFLD) and Non-Alcoholic Steatohepatitis (NASH). Since the mechanisms of this event have not been fully characterized, we aimed to analyze the fibrogenic stimuli in a new *in vitro *model of NASH.

**Methods:**

HuH7 cells were cultured for 24 h in an enriched medium containing bovine serum albumin and increasing concentrations of palmitic and oleic acid at a molar ratio of 1:2 (palmitic and oleic acid, respectively). Cytotoxic effect, apoptosis, oxidative stress, and production of inflammatory and fibrogenic cytokines were measured.

**Results:**

FFA induces a significant increment in the intracellular content of lipid droplets. The gene expression of interleukin-6, interleukin-8 and tumor necrosis factor alpha was significantly increased. The protein level of interleukin-8 was also increased. Intracellular lipid accumulation was associated to a significant up-regulation in the gene expression of transforming growth factor beta 1, alpha 2 macroglobulin, vascular endothelial growth factor A, connective tissue growth factor, insulin-like growth factor 2, thrombospondin 1. Flow cytometry analysis demonstrated a significant increment of early apoptosis and production of reactive oxygen species.

**Conclusions:**

The exposure of hepatocytes to fatty acids elicits inflammation, increase of oxidative stress, apoptosis and production of fibrogenic cytokines. These data support a primary role of FFA in the pathogenesis of NAFLD and NASH.

## Background

Non-alcoholic fatty liver disease (NAFLD) is a common disease associated to obesity and increased visceral fat. This condition has the potential to develop hepatic fibrosis and end-stage liver disease, and is associated with several non-hepatic related complications [1]. Liver fibrosis is a complex event that could be conceptually divided in three phases: initiation, perpetuation and resolution: each of these phases has specific pathways. Classically, the hepatic stellate cell and immune cells are the main players in the fibrotic process [2] but the role of the hepatocyte is also important, mainly in the initiation phase due to production of several fibrogenic stimuli, particularly reactive oxygen species (ROS) [3] and apoptosis [4]. Recently it has been described the production by the hepatocyte of several cytokines involved in the fibrogenesis, and *in vitro *studies with liver cells exposed to toxic amounts of free fatty acids (FFA) demonstrated that this condition could promote and inflammatory and fibrogenic response [5].

The hepatocyte-mediated fibrogenic response is regulated by cytokines involved in proliferation (as the transforming growth factor beta 1 [TGFβ1], alpha 2 macroglobulin [Α2M], vascular endothelial growth factor A [VEGFA], connective tissue growth factor [CTGF], and insulin-like growth factor 2 [IGF2]) and in the regulation of apoptosis (as the nerve growth factor [NGF], and thrombospondin 1 [THBS1]) [2]. In spite of its importance, the fibrogenic response has not been fully explored in NAFLD, and it is also unknown if non toxic amounts of fatty acids are able to induce an inflammatory and fibrogenic response in the hepatocytes.

The aim of this study was therefore to analyze the production of fibrogenic stimulus in a new *in vitro *model of non-alcoholic steatohepatitis (NASH).

## Methods

### Chemicals

Cell culture medium Dulbecco's modified Eagle's high glucose medium (DMEM) (ECB7501L), L- Glutamine (ECB3000D), Penicillin/Streptomycin (ECB3001D), were obtained from Euro-clone (Milan, Italy). Bicinconinc acid solution-kit (B9643); bovine albumin Cohn V fraction (A4503); dimethyl sulphoxide (DMSO) (D2438); fetal bovine serum (F7524); Hoechst 33258 (B1155); hydrogen peroxide (H1009); 3-(4,5 dimethylthiazol-yl-)-2,5-dipheniltetrazoliumbromide (MTT) (M2128); N-acetyl-L-cysteine (NAC) (A9165); Nile Red (N3013); oleic acid (C18:1) (O1008); palmitic acid (C16:0) (P0500); paraformaldehyde (P6148), phosphate-buffered saline (PBS) (D5652); propidium iodide (PI)(P4170) and Tri-Reagent (T9424) were from Sigma Chemical (St.Louis, MO, USA). iScript^™ ^cDNA Synthesis kit (170-8890) and iQ SYBR Green Supermix (170-8860) were purchased from Bio-Rad Laboratories (Hercules, CA, USA). 2',7'-dichlorodihydrofluorescein diacetate (H_2_DCFDA) (D399) was obtained from Molecular Probes (Milan, Italy). Human annexin V-FITC Kit (BMS306FICE); Instant ELISA kit interleukin (IL)-6, IL-8 and tumor necrosis factor (TNF)-alpha (BMS213INSTCE; BMS204/3INSTCE; BMS223INSTCE respectively) were purchased to Bender MedSystems GmbH (Vienna, Austria)

### Cell culture and FFA treatment

Hepatoma derived cell line HuH7 (JHSRRB, Cat #JCRB0403) were obtained from the Health Science Research Resources Bank (Osaka, Japan). Cells were grown in DMEM high glucose medium supplemented with 10% v/v fetal bovine serum, 2 mM L-Glutamine, 10,000 U/mL penicillin and 10 mg/mL streptomycine at 37°C under 5% CO_2_, in a 95% humidified atmosphere. For the treatment, palmitic and oleic acid 0.1 M stock solutions were prepare by dissolving FFA in DMSO. The cells were exposed for 24 h to increasing concentrations of a fresh mixture of exogenous FFA (100; 200; 400; 600 and 1200 μM) in molar ratio 1:2 palmitic:oleic respectively. Since albumin concentration is an important factor in determining the concentration of available FFA, the FFA were complexed with bovine serum albumin at a 4:1 molar ratio taking into consideration the albumin concentration already present in the medium due to the FBS supplementation. The experimental doses were determined in advance by performing dose curves and by assessing cell viability which was always higher than 85% as compared to control cells (100% viability) treated with the equivalent concentration v/v of the vehicle (DMSO).

### MTT assay

Cell viability was assessed using the MTT colorimetric assay. When taken up by living cells, MTT is converted from a yellow to a water insoluble blue-colored precipitate by cellular dehydrogenases [6]. Briefly, 4 × 10^4 ^cells/cm^2 ^were plated in 24 well dish and allowed to adhere overnight. The following day the cells were treated as described above. After 24 h of treatment, the medium was removed and the treatment was followed by addition of 0.5 mg/mL of MTT and incubation at 37°C for 1 h. The medium was then removed, the cells were lysed and the resulting blue formazan crystals were solved in DMSO. The absorbance of each well was read on a microplate reader (Beckman Coulter LD 400 C Luminescence detector) at 570 nm. The absorbance of the untreated controls was taken as 100% survival. Data are expressed as mean ± SD of three independent experiments.

### Fluorimetric determination of intracellular fat content - Nile red staining

Intracellular fat content was determined fluorimetrically based on Nile Red staining, a vital lipophilic dye used to label fat accumulation in the cytosol [7,8]. After 24 h of FFA exposure, adherent monolayer cells were washed twice with PBS and detached by tripsinization. After a 5 min centrifugation at 1500 rpm, the cell pellet was resuspended in 3 mL of PBS and incubated with 0.75 μg/mL Nile red dye for 15 min at room temperature.

Nile red intracellular fluorescence was determined by flow cytofluorometry using a Becton Dickinson FACSCalibur System on the FL2 emission channel through a 585 ± 21 nm band pass filter, following excitation with an argon ion laser source at 488 nm [9]. Data were collected in 10,000 cells and analyzed using Cellquest software from BD Biosciences (San Jose, CA, USA).

### Intracellular lipid droplets analysis by fluorescence microscopy

HuH7 cells were seeded in a coverslip and exposed to FFA for 24 h as previously described. Cells were then washed with PBS twice, and fixed with 3% paraformaldehide for 15 min. Intracellular neutral lipids were stained with Nile Red (3.3 μg/mL) for 15 min. Cell nuclei were stained with Hoescht 33258 dye for 15 min. All the staining procedure was carried out at room temperature by protecting the samples from the direct light. Images were acquired with an inverted fluorescence microscope (Leica DM2000, Wetzlar Germany)

### Extraction of RNA and cDNA synthesis

After the treatment, the medium was removed, cells were washed twice with PBS and after centrifugation, total RNA isolated using Tri-Reagent kit according to manufacturer's instructions. Briefly, cells were lysed with the reagent, chloroform was added and cellular RNA was precipitated by isopropyl alcohol. After washing with 75% ethanol, the RNA pellet was dissolved in nuclease-free water and stored at -80°C until further analysis. RNA was quantified spectrophotometrically at 260 nm in a Beckman Coulter DU^®^730 spectrophotometer (Fullertone, CA, USA). The RNA purity was evaluated by measuring the ratio A260/A280, considering RNA with appropriate purity those showing values between 1.8 and 2.0; its integrity was evaluated by gel electrophoresis. The integrity of RNA was assessed on standard 1% agarose/formaldehyde gel. Isolated RNA was resuspended in RNAse free water and stored at -80°C until analysis. Total RNA (1 μg) was reverse transcribed using iScript^™ ^cDNA Synthesis kit BioRad according to manufacturer's instructions. Retrotranscription was performed in a Thermal Cycler (Gene Amp PCR System 2400, Perkin Elmer, Boston, MA, USA) in agreement with the reaction protocol proposed by the manufacturer's: 5 min at 25°C (annealing), 45 min at 42°C (cDNA synthesis), and 5 min at 85°C (enzyme denaturation).

### Real time quantitative PCR

Real Time quantitative PCR was performed in *i*-Cycler IQ; 18S and β-actin were used as housekeeping genes. All primer pairs were synthesized by Sigma Genosys Ltd. (London Road, UK) and were designed using the software Beacon Designer 7.51 (PREMIER Biosoft International, Palo Alto, CA, USA). Primer sequences and references are specified in Table 1. PCR amplification was carried out in 25 μL reaction volume containing 25 ng of cDNA, 1x iQ SYBR Green Supermix [100 mM KCl; 40 mM Tris-HCl, pH 8.4; 0.4 mM each dNTP; 50 U/mL iTaq DNA polymerase; 6 mM MgCl2; SYBR Green I; 20 nM fluorescein; and stabilizers] and 250 nM gene specific sense and anti-sense primers and 100 nM primers for 18S. Standard curves using a "calibrator" cDNA (chosen among the cDNA samples) were prepared for each target and reference gene. In order to verify the specificity of the amplification, a melt-curve analysis was performed, immediately after the amplification protocol. Non-specific products of PCR were not found in any case. The relative quantification was made using the Pfaffl modification of the ΔΔCt equation, taking into account the efficiencies of individual genes. The results were normalized to 18S and β-actin, the initial amount of the template of each sample was determined as relative expression versus one of the samples chosen as reference (in this case the control sample) which is considered the 1x sample. Results reported are the mean ± SD expression of at least 3 different determinations for each gene.

**Table 1:** Set of primers used in Real-time quantitative PCR

Gene name	Accession number	Forward	Reverse
Interleukin L6	NM_000600	ACAGATTTGAGAGTAGTGAGGAAC	GGCTGGCATTTGTGGTTGG
Interleukin 8	NM_000584	GACATACTCCAAACCTTTCCAC	CTTCTCCACAACCCTCTGC
Tumor necrosis factor alpha	NM_000594	GTGAGGAGGACGAACATC	GAGCCAGAAGAGGTTGAG
Transforming growth factor beta	NM_000660	GCAACAATTCCTGGCGATACC	CTCCACGGCTCAACCACTG
Alpha 2 macroglobulin	NM_000014	AACCAGGACAAGAGGAAGGAAG	AGATAAGCGAGGAGCACATAGG
Vascular endothelial growth factor A	NM_003376	TCGCTTACTCTCACCTGCTTC	TTCCAACAATGTGTCTCTTCTCTTC
Nerve growth factor	NM_002506	AGGAGCAAGCGGTCATCATC	TCTGTGGCGGTGGTCTTATC
Connective tissue growth factor	NM_001901	CGAGGAGTGGGTGTGTGAC	CAGGCAGTTGGCTCTAATCATAG
Thrombospondin 1	NM_003246	ACCAACCGCATTCCAGAGTC	CCGCACAGCATCCACCAG
Insulin-like growth factor 2	NM_000612	CGCTGCTACCGCCATCTC	GTCCCTCTGACTGCTCTGTG

### IL-6, IL-8 and TNF-alpha release

The cytokine released in the culture media was determined by Instant ELISA (Bender MedSystems GmbH; Vienna Austria) according to manufacturer's instructions. Cells were cultured and treated as previously described. The culture media was collected after 24 h, possible contamination of cellular fractions was eliminated by centrifugation and the test performed in cell-free supernatant. According to the manufacturer the lowest detection limits were: 0.92 pg/mL for IL-6, 1.3 pg/mL for IL-8 and 1.65 pg/mL for TNF-alpha.

### Intracellular ROS generation by H_2_DCFDA

Intracellular ROS generation was measured by the use of the cell permeable fluorogenic substrate H_2_DCFDA. This non fluorescent probe is easily taken up by cells and, after intracellular cleavage of the acetyl groups, is trapped and may be oxidized to the fluorescent compound 2',7'-dichlorofluorescin (DCF; the monitored fluorophore) by intracellular ROS [10]. Cells were exposed to FFA for 24 h and after the treatment, adherent cells were washed with PBS, and loaded with 10 μM H_2_DCFDA for 15 min at 37°C. The fluorescence was measured by using a Spectrofluorometer Jasco FP-770 (Excitation wavelength 505 nm and Emission 525 nm). Fluorescence was normalized by the μg of protein assessed by bicinconinic acid protein assay [11] and expressed as AU/μg of protein. The intracellular ROS production was also assessed by flow cytometric analysis by using Becton Dickinson FACSCalibur System. Briefly, attached cells were harvested by tripsinization, washed in PBS by centrifugation, and incubated with 5 μM H_2_DCFDA for 30 min at 37°C in PBS, to exclude hydrogen peroxide generation in phenol red containing media. The cells were washed in PBS and the pellet was suspended in 500 μL of PBS. PI staining was performed to assess non viable cells. Cells within a negative control gate for PI fluorescence were back-gated (FL2) and the fluorescence histogram for H_2_DCFDA (FL1) was applied for the living cells [12]. Considering the potential anti-oxidant effect of albumin [13], the albumin concentration was kept the same in the controls and in the treated samples. Cells exposed to 400 μM hydrogen peroxide were considered as positive control. In order establish if the ROS production induced by FFA could be reversed by an antioxidant agent, cells were co-treated with FFA and 300 μM n-acetylcysteine (NAC). The optimal NAC concentration was established by performing dose curve analysis by MTT, at the used concentration cell viability was unaltered (*data not shown*). Data were collected for 10,000 cells and analyzed using CellQuest software from BD Biosciences (San Jose, CA).

### Assessment of apoptosis

During apoptosis, phosphatidyl serine is exposed from the inner to the outer portion of the membrane and becomes available to bind to the Annexin-V/FITC conjugate. Accordingly, only cells prone to apoptosis will stain positive for Annexin-V/FITC [14]. The cells were harvested by gentle tripsinization which was inactivated by adding medium with 10% FBS. Cells were washed, the pellet suspended in the incubation buffer (10 mM Hepes/NAOH, pH 7.40, 140 mM NaCl, 5 mM CaCl_2_) adjusting cell density to 5 × 10^6 ^cell/mL, and 1 × 10^6 ^cells were incubated with 5 μL Annexin V-FITC and 5 μL PI for 10 min at room temperature. The cells were diluted with the incubation buffer to a final volume of 600 μL and analyzed by flow cytometer using 488 nm excitation and 515 nm band-pass filter for fluorescein detection (FL1) and a filter > 600 nm for PI detection (FL2). Data were collected for 10,000 cells and analyzed using CellQuest software from BD Biosciences (San Jose, CA).

### Statistical analysis

Unless otherwise indicated all the data are expressed as mean ± standard deviation of three independent experiments (biological replicates). Differences between groups were compared by using Student's *t *test. The level of significance was set at a P-value of 0.05.

## Results

### Toxicity of the model

The addition of increasing concentrations of FFA (palmitic/oleic 1:2 molar ratio) did not affect the cell viability measured by MTT Test (data not shown). To assess the effect of the vehicle in which FFA were solubilized, cells were also treated with the equivalent concentration of DMSO of each FFA concentration. In both cases viability was not significantly reduced (10-15%) after 24-h incubation even at the DMSO concentration used with the highest FFA concentrations used (1200 μm).

### Intracellular fat overload

**Figure 1:**
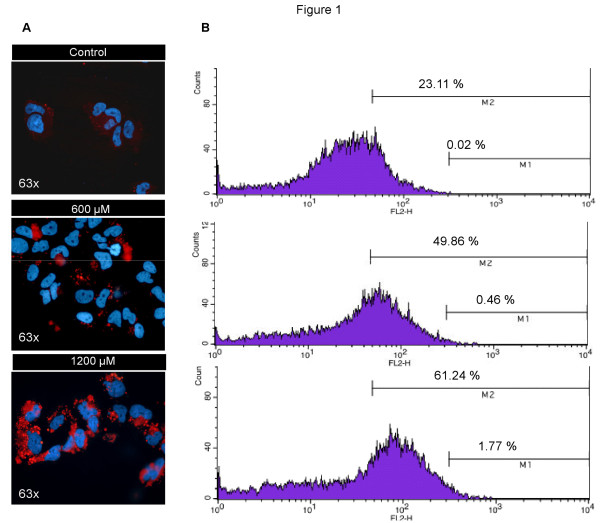
**Dose dependent intracellular fat accumulation**. Cell exposure to 600 and 1200 μM FFA for 24 h. Dose dependent intracellular fat accumulation evidenced by Nile Red staining assessed by fluorescence microscopy (A) and flow cytometry measured in 1 × 10^4 ^cells (B), M1 represents the percentage of events above the maximum value of fluorescence and M2 represents the percentage of events above the median value of fluorescence.

The content of intracellular lipid droplets was determined by Nile Red staining. Cell exposure to 600 and 1200 μM FFA for 24 h induced a dose-dependent fat accumulation. Microscopic images (Figure 1A) showed the presence of cytoplasmic lipid droplets. This pattern was particularly evident after the exposure at 1200 μM FFA where the quantity and size of the droplets increased as compared with that seen with the lower dose. This qualitative data was confirmed by flow cytometry where the presence of cytoplasmatic lipids induced a shift in the median of the fluorescence peak (Figure 1B). After the treatment with FFA, a clear increase of events was observed both in M1 and M2 regions, where M1 represents the percentage of events above the maximum value and M2 the percentage of events above the median value of fluorescence. The total mean fluorescence shows a dose dependent accumulation of FFA (Table 2).

**Table 2:** Intracellular fat accumulation assess by flow cytometry by Nile Red staining.

	*Folds of relative fluorescence vs. control*	*P-value*
Control	1.00 ± 0.20	
600 μM	2.00 ± 0.21	< 0.05
1200 μM	2.50 ± 0.04	< 0.05

The data represent the mean ± SD of three independent experiments.

### Inflammatory cytokines mRNA expression

**Figure 2:**
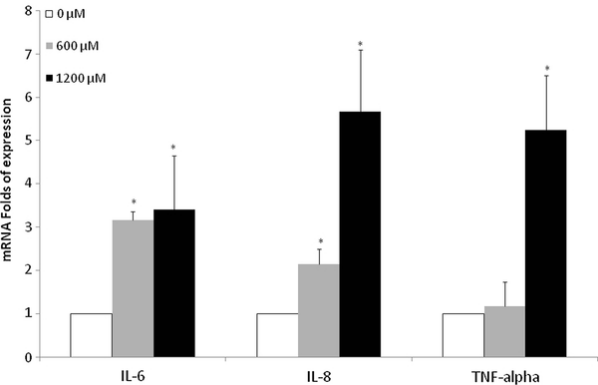
**Inflammatory cytokines mRNA expression**. Cells were cultured and treated with 600 and 1200 μM FFA for 24 h. mRNA expression of IL-6, IL-8 and TNF-alpha in HuH7 cells vs. control. * *P *< 0.05 versus control (0 μM).

The role of inflammatory cytokines in the response of liver cells to several injuries has been described [15]. Figure 2 shows the gene expression of IL-6, IL-8 and TNF-alpha after 24 h of FFA-exposure. All the 3 cytokines were significantly over-expressed as compared to cells treated with the vehicle. However while IL-6 showed a 3-fold increase with no difference between the two doses used, IL-8 showed a clear dose-dependency with a 3-fold difference moving from 600 μM to 1200 μM. Conversely, no increment was observed for TNF-alpha at 600 μM while exposure to 1200 μM resulted in a 5-fold up-regulation in the gene expression.

### Cell release of IL-6, IL-8 and TNF-alpha in the culture medium

**Figure 3:**
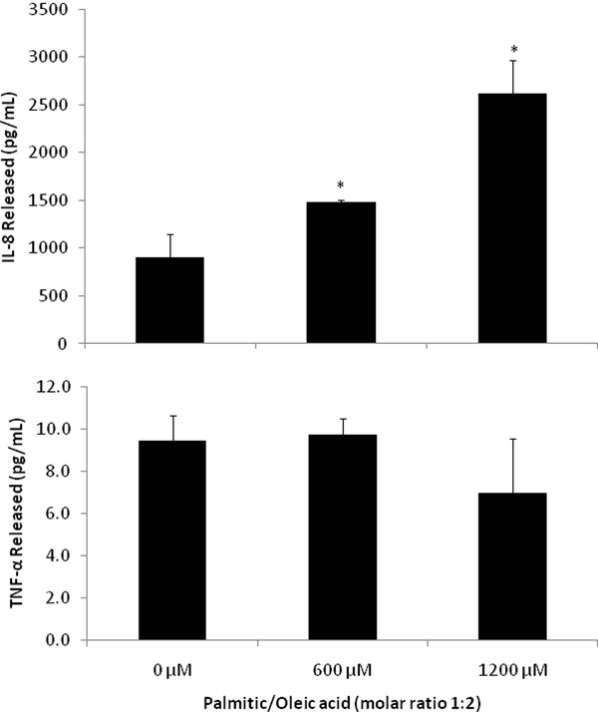
**Inflammatory cytokines protein production**. Inflammatory cytokines present in the supernatant of HuH7 cells after 24 h of the treatment was significantly increased only for IL-8 at both experimental doses, TNF-alpha levels were unchanged, and the levels of IL-6 were lower than the assay sensitivity. * *P *< 0.05 versus control (0 μM).

To investigate if the up-regulation in the mRNA expression was associated to an increased cytokine cell production, the cytokine release into the culture media was also assessed. In line with the data obtained in the mRNA expression (Figure 2), the production of IL-8 was increased in a dose-dependent fashion at both 600 μM (1484 ± 14 vs. 900 ± 246 pg/mL, *p *< 0.05) and 1200 μM (2613 ± 354 vs. 900 ± 246 pg/mL, *p *< 0.05) of FFA (Figure 3). On the contrary and differently from the gene expression, no significant changes were observed for TNF-alpha at any of the experimental concentrations. In line with previous studies [16], IL-6 level was lower than the sensitivity of the method.

### Gene expression of fibrogenic cytokines

**Figure 4:**
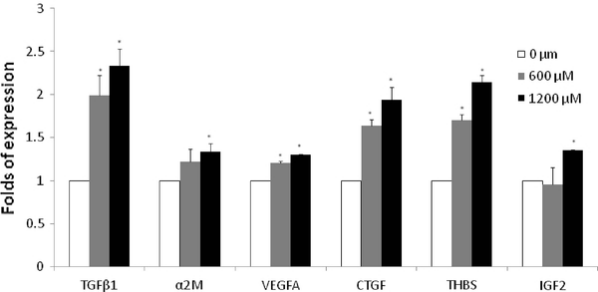
**Fibrogenic cytokines mRNA expression**. Fibrogenic cytokines mRNA expression in HuH7 cells after 24 h exposure at 600 and 1200 μM FFA. * *P *< 0.05 versus control (0 μM).

The involvement of hepatocytes on fibrogenesis has been previously described, and the first phase of fibrosis seems to be mediated by many cytokines [2,16]. To analyze the effect of FFA in our *in vitro *model, a series of cytokines reported as marker of fibrosis in NAFLD [17] and in other liver diseases [18] were assessed. As shown in Figure 4, the intracellular lipid accumulation was associated with a significant up-regulation in TGFβ1, A2M, VEGFA, CTGF, THBS and IGF-2 mRNA. Among these cytokines, the highest increment in gene expression was observed for TGFβ1 (1.99 ± 0.23 and 2.33 ± 0.19 folds of expression vs. control for 600 and 1200 μM, respectively). The same pattern of expression was also observed for all the other cytokines with the exception of a non significant increase for A2M and IGF2 at the lower FFA concentration.

### Intracellular ROS generation

Cells treated with 1200 μM FFA showed a 1.38 ± 0.12 (*p *< 0.05) folds of increase in ROS generation vs. untreated control (Additional file 1: Figure S1 upper panel). The ROS generation was similar to that observed in positive control cells exposed to 400 μM hydrogen peroxide for 3 h (1.48 ± 0.22 folds of increase, *p *< 0.05). When cells treated wither with FFA or hydrogen peroxide were concomitantly exposed to 300 μM of NAC, the ROS level decreased to 1.22 ± 0.12 and 1.07 ± 0.16 (P = NS) fold respectively, indicating that the addition of NAC was able to reverse the alteration in redox state induced by both FFA treatment and hydrogen peroxide. The increment in ROS in FFA treated cells was also confirmed by flow cytometry analysis (333 vs.375 AU respectively) (Additional file 2: Figure S2 lower panel).

### Assessment of apoptosis

**Figure 5:**
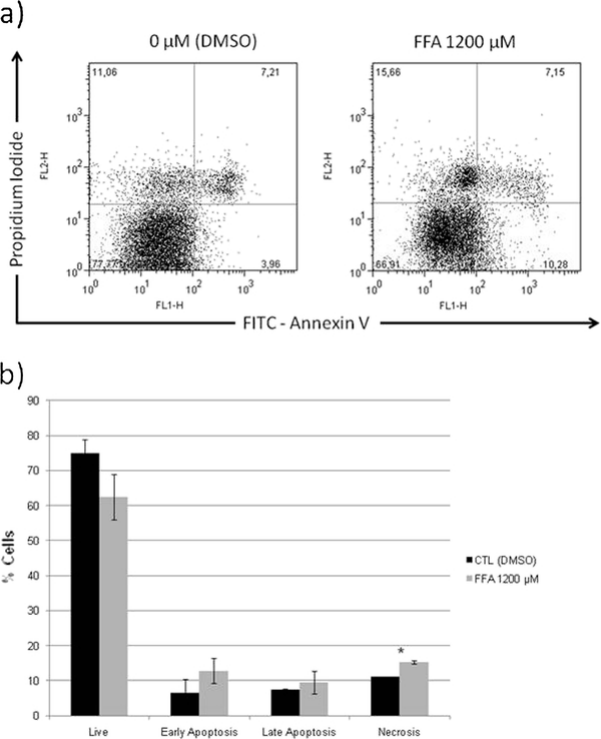
**Apoptosis induction secondary to fatty acids toxicity**. FFA treatment increase apoptosis in hepatic cells (HuH7). There is a reduction in the live cells, and in consequence an increase number of cells in early-, late-apoptosis and necrosis with the FFA treatment. The results are presented as percentage compared to control and represent the mean ± SD, of at least three independent experiments (A). Representative dotplot graph of control and 1200 μM FFA treated cells stained with Annexin V and PI (B).* *P *< 0.05 versus control (0 μM).

HuH7 cells were stained with Annexin V and PI to detect cells with disrupted membrane. According to the cell staining, cells were categorized as: 1) live cells negative for both annexin V-FITC and PI; 2) early apoptotic cells, positive for annexin V-FITC and negative for PI; and 3) late apoptotic cells, positive both for annexin-V-FITCH and PI; cells positive only for PI were considered as necrotic. As shown in Figure 5A 1200 μM of FFA induce an increase in the percentage of cells in early apoptosis (6.2%), late apoptosis (2.1%) and a 4.23% increase in the necrotic population (Figure 5B).

## Discussion

Previous studies showed that the hepatocyte plays a key role in the initiation of fibrosis, and that these events are linked to inflammation, oxidative stress [19] and apoptosis [2]. Studies carried out *in vivo *[20] and *in vitro *models [4] reported the role of several cytokines produced by the hepatocyte in the activation of hepatic stellate cells [21]. More recently extensive evidence was provided on the events associated with the hepatic lipotoxicity [22] with the hepatocyte as the main target of the so called "lipoapoptosis" [23]. In the present work we describe a novel *in vitro *model able to mimic most of the processes observed *in vivo *during the initiation of hepatic fibrosis. A well differentiate hepatic cell line (HuH7) was exposed to different concentrations of a mixture of saturated and non-saturated FFA similar to those found in humans [24]. In addition to the concentration of FFA, the albumin:FFA molar ratio was set to 1:4 [25], since this ratio is important to determine the concentration of the free species of FFA. Moreover, the addition to albumin increases the stability and solubility of FFA in culture medium [26]. With this experimental setup, we observed a dose-dependent intracellular fat accumulation which was not associated to a significant reduction in cell viability, at least for the time frame investigated (24 h). These results point to the conclusion that the hepatic cell line used is able to accumulate FFA and the increased fat content is not associated to a significant impairment of the cell integrity, in line with clinical and *in vivo *experimental data [27]. Similar results were also observed by using no tumoral immortalized human hepatocytes (IHH) [28], even though at a FFA concentration of 1200 μM the intracellular fat accumulation and the inflammatory response was consistently higher and the cell viability significantly reduced (data not shown) [29]. One possible reason for this discrepancy is that the increased FFA susceptibility would be due to the immortalization process, which may introduce alterations in the lipid metabolism.

The importance and novelty of our model is the possibility to measure gene induction and protein expression of the cytokines involved in the pathogenesis of NAFLD [15]. Intracellular FFA accumulation was associated with an inflammatory response elicited by an increased gene expression of IL-6, IL-8 and TNF-alpha, and increased IL-8 protein release. This inflammatory response has been described as a recruitment stimulus and inductor of insulin resistance [25,30]. The increase in the gene expression was rather substantial (3 to 5 folds) for all the 3 cytokines explored at the highest FFA concentration used (1200 μM) indicating that the inflammatory reaction of the hepatocyte is rapid (24 h) and consistent. The behavior of the 3 cytokines is however, different. While the increment in gene expression for both IL-6 and IL-8 was dose-dependent, this was not true for TNF-alpha in which only the highest dose of FFA was able increase the expression. The difference pattern among the 3 cytokines was also confirmed by the amount of cytokines release into the medium. While the IL-8 release showed a clear dose-dependency in line with the gene expression, this was not the case for TNF-alpha. A different response has been reported previously in humans and *in vitro *models, and several hypothesis were made to explain the differential temporal pattern of RNA expression and protein production [31].

As mentioned before, while it is known that liver cells are directly involved in fibrogenesis [2,16], the extent and the timing of this process is still largely unknown. The increased expression of TGFβ1, CTGF and THBS shows that the hepatocyte is able to produce effectors for endothelial and hepatic stellate cells. The up-regulation of a set of genes able to induce fibrosis has been described in other chronic liver diseases [2]. In our model several factors involved in apoptosis regulation, extracellular matrix production, and hepatic stellate cells activation were up-regulated. Albeit many responses were observed in this model, inflammation and induction of apoptosis seem to be the most significant. Apoptosis is considered one of the most important activator of hepatic stellate cells [32]. FFA induce an increased production of alpha-smooth muscle actin, TGFβ1, tissue inhibitor of metalloproteinase 1, and anti-apoptotic proteins [33]. However some paradoxical effects has been described, as the reduction in the collagen 1α expression with the addition of FFA to hepatic stellate cells culture media [34]. In line with this is the demonstration in our study that FFA increase the number of early apoptotic cells. Human NAFLD is associated with oxidative stress, and the resultant lipid peroxidation may determine the transition from simple steatosis to NASH [35]. In our *in vitro *model, FFA treatment induces an increased content of intracellular ROS similar to that produced by the exposure of the cells to a known oxidative agent as hydrogen peroxide. Of notice was the observation that the increased ROS generation following intracellular FFA accumulation was almost completed blunted by co-treating the cells with a known antioxidant agent such as NAC. These data are in agreement with a recent study [19] which suggested that not the production of ROS *per se*, but rather the insufficient or depleted antioxidant defenses is one of the mechanisms associated with the progression of the disease.

One of the main limitation of this study is the use of a HuH7 cell line derived from a well differentiated liver carcinoma [35]. Although these cells do show several characteristics of normal hepatocyte [36], the neoplastic derivation calls for caveats in exporting the observation to the much more complex *in vivo *situation. Despite the intrinsic limitations of *in vitro *studies [27], this novel approach confirm the active participation of the hepatocytes in the damage observed in NAFLD subjects. In addition the model may be also relevant as the hepatocyte has autocrine and paracrine effects on the other resident liver cells [37]. This paracrine relationship is important not only to understand the fibrosis related process, but may be useful to identify candidates for new therapeutic approaches [35]. This is particularly important in NAFLD where few options are effective.

This manuscript is a proof of concept regarding the effects of lipotoxicity in hepatocytes, focused on those factors involved in the fibrogenic stimuli, and attempts to provide additional support for the NAFLD potentiality in developing fibrosis and related complications. It also highlights the independent role of fatty acids in NAFLD. Although the advantages and disadvantages of an *in vitro *model, the main advantage of such an approach is the possibility to dissect the different aspects involved in the pathogenic process in NAFLD, potentially contributing to the improvement of the current diagnostics and therapeutic strategies.

## Conclusions

In conclusion this study demonstrates that FFA induce a wide response on the hepatocyte, ranging from inflammation, increase of oxidative stress, apoptosis and the production of fibrogenic cytokines. It also shows a primary role of FFA in the pathogenesis of NAFLD.

## Abbreviations

NAFLD: Non-alcoholic fatty liver disease; ROS: Reactive oxygen species; FFA: Free fatty acids; TGFβ1: Transforming growth factor beta 1; Α2M: Alpha 2 macroglobulin; VEGFA: Vascular endothelial growth factor A; CTGF: Connective tissue growth factor; IGF2: Insulin-like growth factor 2; NGF: Nerve growth factor; THBS1: Thrombospondin 1; DMEM: Dulbecco's modified Eagle's high glucose medium; DMSO: Dimethyl sulphoxide; MTT: 3-(4,5 dimethylthiazol-yl-)-2,5-dipheniltetrazoliumbromide; NAC: N-acetyl-L-cysteine; PBS: Phosphate-buffered saline; PI: Propidium iodide; H2DCFDA: 2',7'-dichlorodihydrofluorescein diacetate; IL: Interleukin; TNF: Tumor necrosis factor.

## Competing interests

No-financial competing interests.

## Authors' contributions

NCC-T. Design experiments, perform experimental work, data interpretation, wrote and approve the manuscript. NR. Design experiments, perform experimental work, data interpretation, wrote and approve the manuscript. CT. Design experiments, data interpretation, wrote and approve the manuscript. All authors read and approved the final manuscript.

## Pre-publication history

The pre-publication history for this paper can be accessed here:

http://www.biomedcentral.com/1471-230X/12/20/prepub

## Supplementary Material

Additional file 1 **Figure S1**. Increased reactive oxigen species in cells treated with fatty acids. Click here for file

Additional file 2 **Figure S2**. ROS generation on HuH7 cells treated with 1200 μM FFA, mixed with N-acetyl-L-cysteine, or hydrogen peroxide. Measured by spectrophotometric quantification of H_2_DCFDA (upper panel) and flow cytometry (lower panel). *P < 0.05 versus control (0 μM). Click here for file
