# Supplementary figures and images for: Effect of intracellular lipid accumulation in a new model of non-alcoholic fatty liver disease
Source: BMC Gastroenterol. 2012 Mar 1;12:20. doi: 10.1186/1471-230X-12-20 (PMC3313845; doi:10.1186/1471-230X-12-20)

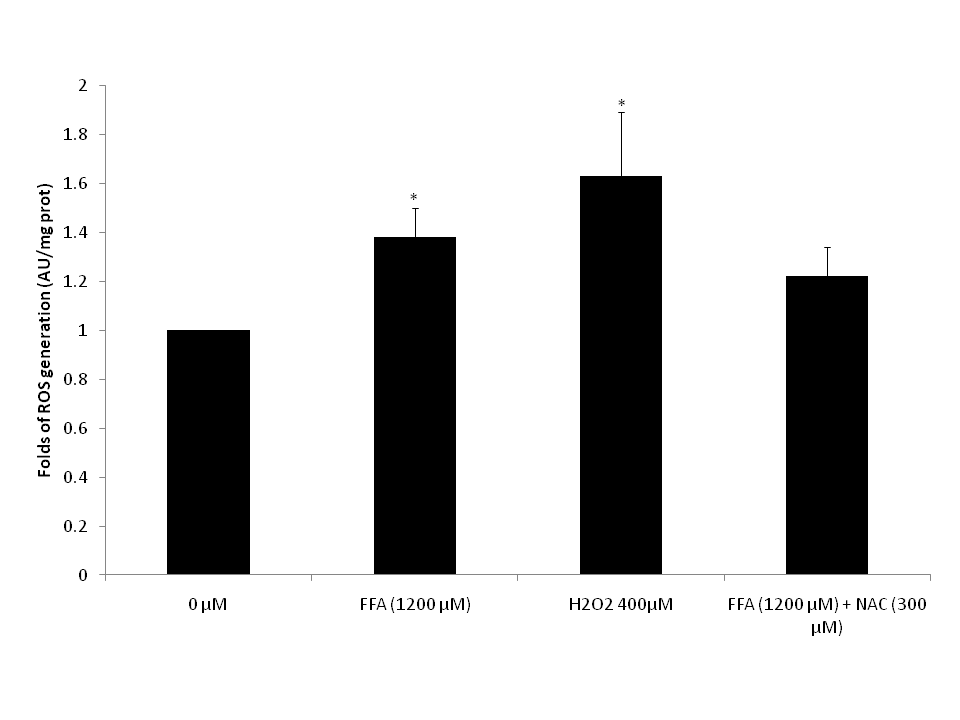

Supplement: Additional file 1 — Figure S1. Increased reactive oxigen species in cells treated with fatty acids. [file 1471-230X-12-20-S1.TIFF]

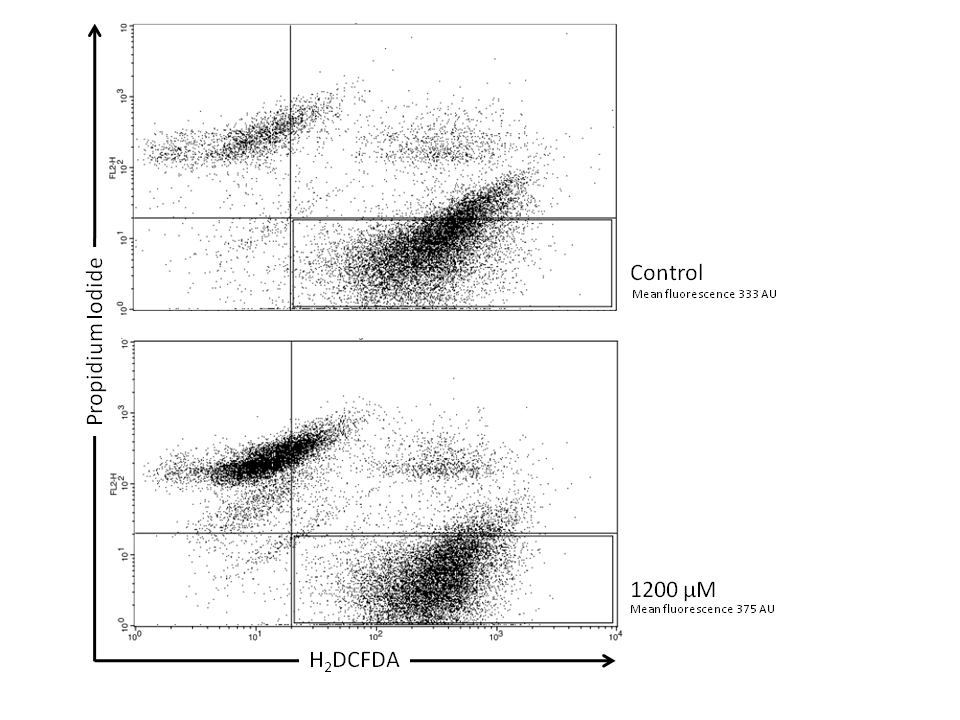

Supplement: Additional file 2 — Figure S2. ROS generation on HuH7 cells treated with 1200 μM FFA, mixed with N-acetyl-L-cysteine, or hydrogen peroxide. Measured by spectrophotometric quantification of H2DCFDA (upper panel) and flow cytometry (lower panel). *P < 0.05 versus control (0 μM). [file 1471-230X-12-20-S2.TIFF]
